# Description of a Pharmacist-Led Mobile Health Clinic to Fill Primary Care Coverage in a Medically Underserved Rural Area

**DOI:** 10.3390/ijerph23050645

**Published:** 2026-05-12

**Authors:** Emily Eddy, Stuart Beatty, David Nau, Karen L. Kier, Michelle Musser, Michael Rush

**Affiliations:** 1 Department of Pharmacy Practice, Ohio Northern Raabe College of Pharmacy, Ada, OH 45810, USA; d-nau@onu.edu (D.N.); k-kier@onu.edu (K.L.K.); m-musser@onu.edu (M.M.); 2 Office of the Dean, Ohio Northern Raabe College of Pharmacy, Ada, OH 45810, USA; s-beatty@onu.edu; 3 HealthWise, Ohio Northern Raabe College of Pharmacy, Ada, OH 45810, USA; m-rush@onu.edu

**Keywords:** mobile clinics, mobile health units, rural health, pharmacist

## Abstract

**Highlights:**

**Public health relevance—How does this work relate to a public health issue?**

- Addresses the growing shortage of primary care providers in the United States, particularly in rural and medically underserved areas, which limits access to essential healthcare services and targets gaps in chronic disease screening and management.
- Responds to barriers to care such as geographic isolation, limited healthcare infrastructure, and social determinants of health, by bringing services directly into communities via a mobile clinic.

**Public health significance—Why is this work of significance to public health?**

- Demonstrates that pharmacist-led mobile health clinics can potentially expand access to primary and preventive care in underserved rural populations.
- Identifies a high burden of undiagnosed or uncontrolled chronic conditions, highlighting the critical need for accessible screening and early intervention services.

**Public health implications—What are the key implications or messages for practitioners, policy makers and/or researchers in public health?**

- Practitioners: Pharmacists can play a central role in delivering primary care services (screening, medication management, education) through collaborative practice models. Policymakers: Support for expanded scope of practice, reimbursement models, and funding for mobile clinics could improve access and reduce rural health disparities.
- Researchers: Further studies are needed to evaluate long-term clinical outcomes, cost-effectiveness, and scalability of the pharmacist-led mobile health clinic model.

**Abstract:**

Objective: Describe a mobile health clinic program led by pharmacists to provide services in a primary care shortage area. Methods: ONU HealthWise is a comprehensive pharmacy service offered by Ohio Northern University Raabe College of Pharmacy with a mobile clinic initiated in 2015. ONU HealthWise is located in an HRSA-designated medically underserved and primary care shortage area and the mobile health clinic visits 11–18 locations monthly plus additional sites for screening or vaccinations. Medical residents from a health-system attend some locations and collaborative practice agreements allow pharmacists to initiate and adjust medications. Student pharmacists rotate through the mobile clinic to gain experiential training toward their Doctor of Pharmacy. The mobile clinic is an integral part of the learning and precepting for ONU HealthWise PGY-1 residents. Results: Over a 12-month period (July 2024–June 2025), the mobile clinic held 148 clinics across 7 rural counties in northwest Ohio. A total of 1265 screenings were conducted at 713 patient encounters (604 unique patients). Of the screenings, 38.1% of blood glucose, 21.6% of cholesterol, and 60.1% of blood pressures were abnormal. All abnormal tests resulted in either a medication adjustment, scheduled follow-up at future mobile clinic, or referral to a provider. Student pharmacists spent more than 3670 h on the mobile health clinic in experiential education. Conclusion: Pharmacists can be an integral healthcare provider by increasing access to primary care services through a mobile health clinic in a medically underserved area. The service provides learners with vital patient experiences.

## 1. Introduction

The United States faces a worsening shortage of primary care physicians (PCPs) driven by an aging population needing more care, a retiring physician workforce, high levels of burnout, and lower reimbursement/salary compared to specialty care. Both the Association of American Medical Colleges (AAMC) and Health Resources and Services Administration (HRSA) project substantial primary care shortages in the coming decade, with HRSA estimating a shortage of 87,150 full-time equivalent primary care physicians in 2037 [1,2]. These workforce shortage projections will result in reduced patient access to care, particularly in rural areas [3].

Rural areas are disproportionately affected by the primary care shortage. Nearly 7.8% of U.S. counties have no primary care physicians, the vast majority in rural counties [4]. Given this shortage, there have been several ways in which the healthcare system has attempted to alleviate the strain. One approach has been the expanded use of physician extenders, such as nurse practitioners and physician assistants. While these professionals help mitigate workforce shortages, their distribution mirrors that of physicians, leaving many rural counties underserved. Another methodology, implemented widely during and after the COVID-19 pandemic, is telehealth-based primary care. Telehealth has improved access for some rural patients; however, it remains an incomplete solution due to persistent barriers such as limited broadband availability, digital literacy challenges, and reduced access among older adults and socioeconomically vulnerable populations [5,6]. Consequently, creative and community-centered solutions remain necessary to improve access to primary care services in rural populations.

Mobile health clinics (MHCs) are an innovative solution to improving healthcare access for vulnerable populations in rural areas. A comprehensive review by Yu and colleagues examined 51 articles related to the strengths and weaknesses of MHCs. The authors concluded that “current literature supports that MHCs are successful in reaching vulnerable populations, by delivering services directly at the curbside in communities of need and flexibly adapting their services based on the changing needs of the target community” [7]. Additionally, the CDC Community Preventive Services Task Force recommends the use of mobile clinics for improving screening and initiation of care for chronic diseases [8].

Yu and colleagues noted that MHCs are highly effective at overcoming geographic and logistical barriers to care by bringing healthcare services to patients. The use of consistent, locally based providers fosters trust and sustained engagement with patients, while the inherent flexibility of MHCs allows for rapid adaptation during public health emergencies [7].

Pharmacists are one of the most accessible healthcare providers in the United States. They are uniquely positioned to address gaps in primary care delivery, particularly in rural and underserved communities, with patients visiting the community pharmacy more often than a primary care provider [9]. In 2011, the Surgeon General of the United States called for the use of pharmacists and pharmacy practice as a way to improve patient and health system outcomes [10]. Pharmacists are trained to manage chronic diseases, provide preventive care, perform medication therapy management, and deliver patient education—services that align closely with core primary care functions. Evidence demonstrates that pharmacist-led interventions improve outcomes for chronic conditions such as hypertension, diabetes, and hyperlipidemia, while reducing healthcare utilization and costs [11].

The CDC Community Preventive Services Task Force specifically recommends pharmacist-led, tailored pharmacy-based interventions to improve medication adherence for the prevention of heart disease and stroke, citing strong evidence of effectiveness [12]. Despite this evidence, pharmacists remain underutilized in many primary care delivery models, particularly within rural settings.

Mobile health clinics provide an ideal platform for integrating pharmacists into team-based primary care delivery. The combination of pharmacist expertise and the mobility of MHCs offers a promising strategy to expand access to preventive services, chronic disease management, and medication optimization in rural communities. A paucity of research on pharmacist-led mobile clinics currently exists which leads to the utility of this descriptive paper on such a model. Describing pharmacist-led care in a mobile clinic setting can provide valuable insights into clinical outcomes, access to care, patient satisfaction and workforce development in a rural population. Such assessment is essential to inform policy, optimize workforce utilization, and support scalable models that address primary care gaps in underserved and rural populations.

## 2. Materials and Methods

### 2.1. Study Design

This descriptive evaluation characterizes the structure, operations, and service delivery model of the ONU HealthWise Mobile Health Clinic (ONUHMHC), with a focus on its capacity to expand access to primary care services within multiple rural and underserved communities. Pharmacists provide care on the ONUHMHC and serve as clinical instructors to student pharmacists, who obtain Introductory and Advanced Pharmacy Practice Experience. Clinical outcomes are tracked from each patient encounter; learning outcomes are measured through student evaluations and pre/post student survey and feedback.

### 2.2. Setting

The Ohio Northern University Raabe College of Pharmacy is an accredited provider of a Doctor of Pharmacy (PharmD) program. Located in the rural village of Ada, Ohio and founded in 1884, the College has a long history of developing pharmacist leaders. ONU HealthWise is the clinical practice network of the Raabe College of Pharmacy. The ONUHMHC operates throughout seven rural counties in northwest and west central Ohio. These counties include areas designated by the Health Resources and Services Administration (HRSA) as medically underserved areas and primary care Health Professional Shortage Areas, reflecting limited provider availability and substantial barriers to timely access to care.

### 2.3. Mobile Clinic Operations

ONUHMHC was initiated in 2015 to deliver community-based primary and preventive care services within rural settings with limited healthcare infrastructure. The clinic provides care at scheduled locations each month across the seven-county service area. Clinic locations are coordinated with community leaders and include community centers, community meals, churches, social service agencies, food distribution services, local businesses, and other community gathering sites. Additional clinic events are conducted as requested to support targeted preventive services, such as disease screening events or community-wide vaccination programs.

Funding for the ONUHMHC is sustained through a diverse and balanced mix of revenue streams designed to support both its clinical services and community outreach mission. These sources include direct medical insurance billing for patient care services, prescription billing through the dispensing of medications, and competitive grant funding that enables program expansion and innovation. In addition, the clinic benefits from corporate sponsorships and charitable contributions from individuals and organizations. At the time of the study, a 38-foot motorcoach served as the mobile care platform, equipped with two examination rooms, a patient intake and education area, point-of-care testing capabilities, and wheelchair-accessible entry. A consistent, recurring schedule within each county supports continuity, community recognition, and improved access for individuals lacking regular primary care.

### 2.4. Clinical Services and Collaborative Practice

Clinical services are pharmacist-led and designed to address preventive care gaps and management of chronic primary care needs in rural populations; review of state and local county health improvement plans drove selection of clinical services and testing offered. Point-of-care testing allows assessment and monitoring of cholesterol (lipids), hemoglobin A1c, blood glucose, blood pressure, skin scope, and bone mineral density. Point-of-care testing results are recorded as normal or abnormal. Abnormal values were individualized for each patient based on clinical presentation and interpreted in accordance with the ADA Standards of Care, the 2017 ACC/AHA hypertension guideline, and the 2018 ACC/AHA dyslipidemia management guideline [13,14,15].

Patients voluntarily present to the clinic for initial care. All patients receive a comprehensive health risk assessment, immunization review, and a comprehensive medication review. Immunizations are available at each location. Patients are provided primary care services and preventive health counseling. Monitoring plans are developed at each visit, and patients are referred for follow-up or scheduled for a visit during a future ONUHMHC stop. All patient encounters are documented, and notes are shared with other providers. The mobile clinic played a crucial role during the COVID-19 pandemic by providing immunizations throughout a 14-county rural region of Ohio as a designated mass vaccination clinic.

Pharmacist providers operate under collaborative practice agreements (CPAs) established with partnering medical providers, granting authority to initiate and modify medication therapy as required per Ohio State Board of Pharmacy regulations. Medical residents from a nearby health system attend selected clinic sites and participate in all aspects of the patient visit, offering patients interprofessional education and access to physician services at some locations.

### 2.5. Student Pharmacist Training

Doctor of Pharmacy students are required to complete introductory pharmacy practice experiences (IPPE) as part of the PharmD curriculum. All PharmD students must complete forty hours of IPPE on the ONUHMHC during one semester in the second or third year of the professional curriculum. Students may elect to complete a month-long (minimum 160 h) advanced pharmacy practice experience (APPE) with ONU Healthwise during the sixth year of the program. ONU HealthWise is a site for two first-year post-graduate (PGY1) pharmacy resident trainees, licensed pharmacists who gain clinical and research experience. Under the supervision of licensed pharmacists including PGY1 pharmacy residents, students participate in patient intake, screening procedures, immunization delivery, medication review, counseling, documentation, and interprofessional communication. This model integrates patient care with workforce development in rural healthcare delivery. A layered learning model, with IPPE students, APPE students, and pharmacy residents, is utilized to allow all learners the opportunity to provide care directly with an additional experience of mentoring and supervising less experienced learners.

## 3. Results

### 3.1. Clinical Outcomes

Over a 12-month period (July 2024 through June 2025), the ONUHMHC program held 148 clinics across 7 counties in rural areas of northwest Ohio, United States. The clinic provided 713 patient encounters for 604 unique patients, of whom 53.97% were women and 32.7% were non-white (Table 1). Repeat visits, referrals, and treatment initiation data were not tracked.

A total of 1265 screening tests were conducted for the 713 patient encounters. The number of tests and the frequency of abnormal tests are reflected in Table 2. The most common screening was of blood pressure, with more than 60% of patients having elevated blood pressure readings. Many individuals were identified with poor glycemic control wherein 38.1% of patients had fingerstick blood glucose results that were elevated and 80.8% of A1c tests performed were above goal.

### 3.2. Education Outcomes

Over the same 12-month period (July 2024 through June 2025), 68 students completed IPPE hours on the ONUHMHC, equating to nearly 1800 h of clinical service. Based on student feedback following the experience, the mean rating for the IPPE experience was 3.92/4 (4 = excellent, 3 = good, 2 = average, 1 = poor). Students strongly agreed the experience was valuable to their learning (mean = 4.88/5), enjoyable (mean = 4.85/5), and allowed them to impact the health of the community (4.83/5). Students strongly agreed that the longitudinal, required experience with the ONUHMHC was valuable (mean = 4.62/5). (5 = strongly agree, 4 = agree, 3 = neutral, 2 = disagree, 1 = strongly disagree).

From July 2024 to June 2025, ONU Healthwise provided a month-long APPE to eleven students; this equates to 1870 h of APPE experience. Average scores on student evaluations of the rotation and preceptor following the experience were 5/5 (5 = strongly agree, 4 = agree, 3 = neutral, 2 = disagree, 1 = strongly disagree).

## 4. Discussion

The described ONUHMHC model demonstrates a viable and impactful strategy for integrating pharmacists into primary care delivery within medically underserved rural communities. The high prevalence of abnormal screening results observed—particularly for elevated blood pressure (60.1%) and poor glycemic control (38.1% elevated blood glucose, 80.8% A1c above goal)—highlights the significant unmet chronic disease management needs of the rural patient population served. And while attendees were self-selected, thus reflecting screening yield among individuals who chose to engage with the mobile clinic and should not be mistaken for a community prevalence estimate, they do provide evidence of uncontrolled disease states in the community. Diabetes, hypertension, and hyperlipidemia increase the likelihood of heart disease, the leading cause of death in the United States. People living in rural areas are more likely to develop complications or premature death from heart disease compared to those in metropolitan areas [16]. By providing accessible, point-of-care testing and pharmacist-led interventions, the ONUHMHC actively identifies, monitors, and facilitates the management of these critical health issues, directly addressing the primary care gap in the region.

The ONUHMHC model uses pharmacists as the primary deliverer of care to patients. Pharmacists’ roles have evolved drastically over the past 30 years from a focus on product dispensing to the provision of direct patient care services. According to the 2022 National Health and Nutrition Examination Survey, 12% of the overall population takes five or more prescription medications [17]. Among people 65 years of age and older, 43% of patients take five or more prescription medications, up from only 33% in 2004 [18]. With a professional doctorate degree, a minimum of 6 years of higher education, and the most didactic hours of any profession dedicated to medication use, pharmacists are an ideal partner to engage in the management of patients with chronic diseases. Multiple organizations, including the US Surgeon General’s office and the National Governors’ Association [10,19] have called on pharmacists to be included in health care teams to improve care and lower costs. A recent study showed this impact, with 61.8% of patients with pharmacist involvement achieving blood pressure control compared to 47.7% without a pharmacist [9].

Within the ONUHMHC, pharmacists are working as part of the healthcare team and using their expertise and accessibility to fill the primary care gaps in rural areas. All identified abnormal results lead to a defined clinical action, such as medication adjustment under Collaborative Practice Agreements (CPAs), scheduled follow-up, or referral to another provider or specialist. This systematic approach ensures that screening translates into tangible steps toward improved health, a crucial component of effective chronic disease management in populations with limited access to consistent care.

The ONUHMHC model was designed on evidence-based principles to use consistent, locally based providers to build trust with rural populations [8]. It brings care to rural communities that lack healthcare resources and provides care to any patient who wants care, overcoming barriers related to location, transportation, or insurance status, minimizing transportation challenges and fostering patient engagement through regular clinician presence. Whenever possible, delays in care are overcome by pharmacists initiating medication therapy and care coordination during a mobile clinic visit. Follow-up can be accomplished through scheduled appointments with the mobile clinic, but can also occur via telehealth with a team of pharmacists and student pharmacists as part of the ONU HealthWise program. This comprehensive approach allows the team to utilize digital health solutions when possible, while allowing patients the ability to call a trusted team from the clinic for questions or advice in between appointments. As a trusted partner in the community and with its ability to drive directly to where patients live, the ONUHMHC can respond to local needs or emergencies. This operational flexibility was demonstrated during the COVID-19 pandemic, where ONUHMHC served as a mass vaccination site, proving its value in rapidly adapting to public health emergencies.

Beyond clinical service, the ONUHMHC serves as a vital component of the rural healthcare workforce training. The implementation of a layered learning model for pharmacists, involving IPPE students, APPE students, and PGY1 pharmacy residents, provides extensive experiential training in direct patient care, interprofessional collaboration, and community outreach. Students rated the experience as highly valuable and reported a positive perception of their contribution to community health. This development of pharmacist expertise in a rural mobile setting is essential for cultivating a future workforce equipped and motivated to practice in underserved areas, while also providing trainees the ability to see firsthand the impact a pharmacist can make in a primary care workforce shortage area.

## 5. Limitations

This study is a descriptive evaluation of a single mobile health clinic program. While the results describe the feasibility and need for clinical services, the lack of a comparator group limits the ability to demonstrate impact on clinical outcomes. The 12-month data captures activity within a specific timeframe and geographic area; generalizability to other regions or different mobile health models may be limited. Clinical patients self-select to obtain services at the mobile health clinic program, which introduces potential bias when evaluating clinical outcomes. Community prevalence can not be extrapolated because of the self-selection bias, but it does allow for evidence that there is an unmet disease state need in the community on some level. Future research should focus on longitudinal patient outcome data and comparative studies to further quantify the impact of pharmacist-led mobile health clinics on long-term disease control and healthcare utilization.

## 6. Conclusions

The ONU HealthWise Mobile Health Clinic model presents a scalable and potentially effective solution to the persistent primary care shortages and healthcare disparities found in rural America. By leveraging the clinical expertise of pharmacists and the flexibility of a mobile platform, the program provides a potential solution to bridge the gap between underserved populations and essential health services. The high volume of abnormal screenings identified during the study period underscores the critical need for such community-based interventions in managing chronic conditions like hypertension and diabetes.

The integration of a layered learning model demonstrates that mobile clinics can serve as a dual-purpose engine for both immediate public health impact and long-term workforce development. By immersing student pharmacists in rural practice, the program fosters the skills and commitment necessary to sustain the future of rural healthcare. Moving forward, continued support for expanded pharmacist scopes of practice and innovative reimbursement models will be essential to replicate this success and ensure that geographic isolation no longer equates to a lack of quality care.

## Figures and Tables

**Table 1:** Patient Demographics.

Patient Demographics	Count (%)
Female	326 (53.97%)
Male	278 (46.03%)
Asian	8 (1.32%)
Black	53 (8.77%)
Native American	6 (0.99%)
White	408 (67.55%)
Multiracial	9 (1.49%)
Declined to state race	120 (19.87%)

**Table 2:** Screening Results.

Test	Count	% Abnormal ^b^
Blood Pressure	589	354 (60.1%)
Blood Glucose (fasting or random)	407	155 (38.1%)
Total cholesterol	134	29 (21.6%)
Triglycerides	134	51 (38.1%)
HDL	134	50 (37.3%)
LDL	134	56 (41.8%)
A1c	52	42 (80.8%)
BMD ^a^	37	14 (37.8%)
Skin Scope	37	5 (13.5%)

^a^. Bone Mineral Density (BMD). ^b^. Individualized for each patient based on clinical presentation and interpreted in accordance with the ADA Standards of Care, the 2017 ACC/AHA hypertension guideline, and the 2018 ACC/AHA dyslipidemia management guideline [13,14,15].

## Data Availability

The original contributions presented in this study are included in the article. Further inquiries can be directed to the corresponding author.
